# Understanding disease mechanisms with models of signaling pathway activities

**DOI:** 10.1186/s12918-014-0121-3

**Published:** 2014-10-25

**Authors:** Patricia Sebastian-Leon, Enrique Vidal, Pablo Minguez, Ana Conesa, Sonia Tarazona, Alicia Amadoz, Carmen Armero, Francisco Salavert, Antonio Vidal-Puig, David Montaner, Joaquín Dopazo

**Affiliations:** Department of Computational Genomics, Centro de Investigación Príncipe Felipe (CIPF), Avda. Autopista del Saler, 16, 46012 Valencia, Spain; BIER, CIBER de Enfermedades Raras (CIBERER), Valencia, 46012 Spain; Present Address: Cancer Epigenetics and Biology Program (PEBC), Bellvitge Biomedical Research Institute (IDIBELL), L’Hospitalet de Llobregat, Barcelona, Spain; Present Address: Structural and Computational Biology, European Molecular Biology Laboratory, Heidelberg, 69117 Germany; Department of Statistics and Operations Research, University of Valencia, Valencia, 46100 Spain; Institute of Metabolic Science - Metabolic Research Laboratories and Department of Clinical Biochemistry, University of Cambridge, Addenbrooke’s Hospital, Cambridge, CB2 0QQ UK; Functional Genomics Node, (INB) at CIPF, Valencia, Spain

**Keywords:** Signaling pathways, Probabilistic model, Disease mechanism, Precision medicine, Disease mechanism, Cancer, Fanconi anemia, Obesity, Stem cells

## Abstract

**Background:**

Understanding the aspects of the cell functionality that account for disease or drug action mechanisms is one of the main challenges in the analysis of genomic data and is on the basis of the future implementation of precision medicine.

**Results:**

Here we propose a simple probabilistic model in which signaling pathways are separated into elementary sub-pathways or signal transmission circuits (which ultimately trigger cell functions) and then transforms gene expression measurements into probabilities of activation of such signal transmission circuits. Using this model, differential activation of such circuits between biological conditions can be estimated. Thus, circuit activation statuses can be interpreted as biomarkers that discriminate among the compared conditions. This type of mechanism-based biomarkers accounts for cell functional activities and can easily be associated to disease or drug action mechanisms. The accuracy of the proposed model is demonstrated with simulations and real datasets.

**Conclusions:**

The proposed model provides detailed information that enables the interpretation disease mechanisms as a consequence of the complex combinations of altered gene expression values. Moreover, it offers a framework for suggesting possible ways of therapeutic intervention in a pathologically perturbed system.

**Electronic supplementary material:**

The online version of this article (doi:10.1186/s12918-014-0121-3) contains supplementary material, which is available to authorized users.

## Background

Precision medicine requires of better ways of defining diseases by introducing state-of-the-art genomic technologies into the diagnostic procedures. A more precise diagnostic of diseases, based on the description of their molecular mechanisms, is critical for creating innovative diagnostic, prognostic, and therapeutic strategies properly tailored to each patient’s necessities [1]. Consequently, a deeper understanding of the consequences that the combined effect of gene activities has over the functionality of the cell has become a major concern in the analysis of genomic data. Moreover, this knowledge is essential to understand how gene activity perturbations account for disease. The analysis of genome-wide expression profiles has produced an unprecedented amount of data on gene activity under a plethora of experimental conditions. Nowadays more than one million of microarray experiments are available, stored in public databases. However, the conversion of such data into a better understanding of the underlying biological processes that explain molecular mechanisms of diseases still remains an open issue. Early attempts to deal with the problem of understanding the collective contribution of gene activities to the definition of phenotypes (e.g. disease) produced a family of algorithms commonly known as single enrichment methods (SEA) [2]. Such methods study over-representations of functional annotations in groups of differentially expressed genes [3-8]. Typical functional annotations used for this purpose are gene ontology (GO) [9], KEGG pathways [10], etc. Later, the gene set enrichment analysis (GSEA) method [11] pioneered another family of more sensitive algorithms based on testing the distribution of functional annotations along a list of genes ranked by differential expression [12-16]. Both approaches consider functional modules as discrete, unstructured entities composed of elements (proteins) of identical importance with respect to the trait analyzed. This is a quite unrealistic assumption that drastically reduces the statistical power in any testing framework [17].

Nevertheless, functional modules with detailed information on their internal structures are available in several repositories such as KEGG [10], Reactome [18], etc. In fact, there is a recent interest in exploiting the information contained in different biological pathways to understand cellular function and disease mechanisms [19]. Recently, a few pathway topology PT-based algorithms have been published [20] that use the internal structure of the pathway to elucidate global activation status for the pathway from gene expression data. Some PT-based algorithms use the pathway structure to compute measurements of coordinated activity (such as correlation, covariance, etc.) among pairs of genes [21]. Other, as the signaling pathway impact analysis (SPIA) [22,23] or the NetGSA [24], infer scores that account for the global activation status of pathways taking into account the relationships among their constituent gene products. Conceptually similar methods, implementing more sophisticated ways of scoring pathway global activity based in the relationships among the corresponding gene products, have also been proposed [25-35].

In most of the methods described above individual values accounting for gene expression are combined, as defined by the internal structure of the pathway, into a global score for the entire pathway. That is, pathways are used as whole functional units in the interpretation of gene expression experiments. However, the biological consequence of the activity of a pathway is not a trivial concept. Stating that a pathway is activated (or deactivated) is not very informative by itself. In fact, partial activation (or deactivation) within the same pathway can have very different (and sometimes opposite) biological implications. For example, the apoptosis signaling pathway can lead to two opposite cell behaviors: apoptosis and degradation (cell death) or antiapoptosis (cell survival) depending on the final protein that receives the signal (see hsa04210 pathway in the KEGG repository). Therefore, depending on their relative location within the pathway, changes in the activation state of one or several proteins can change (and even reverse) the cell behavior. Some of the PT-based methods use values of differential gene expression within the context of the pathways to calculate scores for pathway sub-structures [28-31,36]. However, such scores have a difficult interpretation for both significant and non-significant parts of the pathway because the differential expression of a gene does not necessarily imply a transition from an active to an inactive state (or vice versa). However, the most serious problem occurs when one or several inhibitors within a pathway present a non-significant differential expression. In this case, its activation state is unknown and, consequently, its effect (normally drastic) on the sub-pathway.

From a completely different angle, other approaches have attempted to model protein signaling networks in detail [37-40]. Such approaches offer a detailed description of the network modeled and have been used to check for inconsistences in the network or for finding new interdependences between their constituent elements. These models were used to study properties of the pathways analyzed [41], to optimize network models using experimental data (protein activations, measured as phosphorylation or other biochemical properties) [37], or to study pathway responses [42], but none of these approaches consider the use of gene expression measurements derived from transcriptomic experiments (probably the most frequently used genomic data in biomedical research during the last decade).

The approach proposed here offers a conceptually new way of understanding the functional consequences of changes in gene activity associated to any comparison of conditions by taking into account the internal structure of the pathways. This structure is used to split up the pathway into stimulus-response sub-pathways, or circuits, which trigger particular cell functionalities. In other words, the method enables the estimation of the probabilities of activation of these cell functionalities. This approach allows overtaking the simplistic view of a pathway as an entity that is either active or inactive as a whole. Here we show how the analysis of these signaling circuits results in a more detailed and realistic description of the functional consequences of gene up-and down-regulations within the context of each pathway. In many cases, the resulting activation/deactivation of individual functionalities within the pathway helps to suggest a causal explanation for disease mechanisms. We illustrate the application of the methodology with gene expression data obtained from various microarray experiments with different conditions and diseases.

## Results and discussion

### Performance of the methodology

As previously commented pathways are multifunctional entities that can trigger a range of different cell functions and it is often meaningless to talk about the activation of a pathway as a whole or relating its global activity to a disease. Opposite to this view, we propose a different method which accounts for the change in the probabilities of signal transmission along the different signaling circuits defined within pathways. Briefly, we first use the values of gene expression to infer probabilities of gene product presence/absence (and, consequently, their potential for transmitting a signal). Then, we use such probabilities to calculate the probability of signal transmission along the circuit, from the receptor protein (that receives the stimulus) to the effector protein (that triggers the response). We take into account that interactions between proteins can be either activations or inhibitions (see Methods for details). If the probability of having the circuit activated for signal transmission is significantly higher in one of the conditions of the comparison then a change in a specific pathway activity is reported as a result. Since some circuits of the modeled pathways might be declared significant simply as a consequence of type I errors (false positives), we carried out a simulation and we also studied a real case in which no differences were expected.

**Figure 1:**
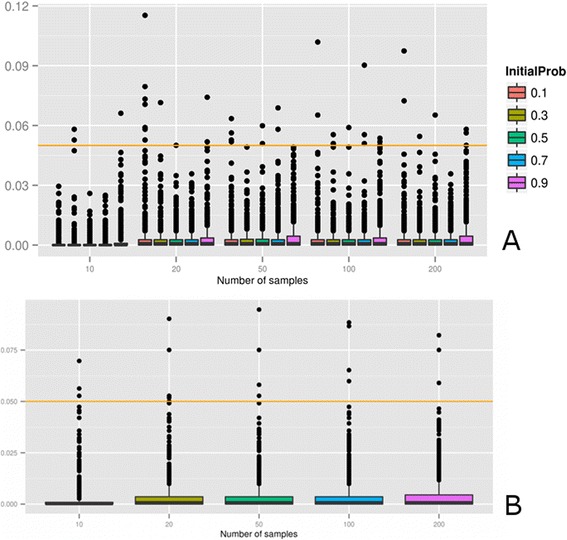
**Analysis of the false discovery rate of the signaling circuit test proposed. A)** Results of the simulation experiment. A total of 1000 datasets, of n = 10, 20, 50 and 100 samples were simulated. All the probes for all the genes in all the nodes of the pathways had the same probability of being activated (0.1, 0.3, 0.5, 0.7 and 0.9). Normal distributed (mean = 0, SD = 0.05 noise was added to each probe). At the conventional p-value of 0.05 chosen for the test, the ratios of false positives are negligible. **B)** Results with a real dataset of pediatric acute myeloid leukemia (AML) which contains gene expression microarray data of 237 samples, which (in principle) should display a similar gene expression profile. We produced 1000 datasets, of n = 10, 20, 50 and 100 samples, by randomly subsampling the 237 samples dataset. Any of the datasets was divided into two equal parts and compared to each other with the test. Again, the number of false positives is negligible.

We first simulate probe datasets with identical probability of being activated, to which normally distributed noise is added (mean zero, and standard deviation 0.05). All the samples would represent pathways whose genes will have a similar activity state and, consequently, should have their signaling circuits in the same activity state. For this reason, no differences should be observed in any comparison. We produced 1000 datasets, of n = 10, 20, 50 and 100 samples with probabilities 0.1, 0.3, 0.5, 0.7 and 0.9 of being activated. Figure 1A shows the results of the simulation. At the conventional p-value of 0.05 chosen for the test the ratios of false positives are negligible (and far below the expected 5%).

We have also used a large dataset of pediatric acute myeloid leukemia (AML) which contains gene expression microarray data of 237 children [43]. In principle all samples should have a similar profile of gene expression, and consequently similar activation states in the signaling circuit. We produce 1000 datasets, of n = 10, 20, 50 and 100 samples, by randomly subsampling the 237 samples. Any of the datasets was divided into two equal parts and compared to each other with the proposed methodology. Figure 1B shows that for any data size (given the p-value of 0.05 used in the test as threshold) the number of false positives is almost inexistent. In fact, some real biological variation that could result in slight circuit activity differences cannot be ruled out. Consequently, some of these very few false positives might, in reality, be true positives as well.

Additionally, two microarray datasets containing cases and controls in a study of breast cancer (GSE27562) [44] and another study of genes with increased expression in AML (GSE9476) [45] were used to check the predictive performance of features consisting of circuit activation values estimated using the proposed method. This predictive performance can be considered representative of a low number of failures in detecting real activations (false negatives) [26,35] and allows us to check for the extent of type II error. We used Support Vector Machine (SVM) [46] for the classification of the samples. The accuracy of the classification obtained was evaluated by ten-fold cross validation [47], using the following parameters: proportion of correct classification (PCC) and the area under the curve (AUC). The results obtained were in: for the breast cancer dataset, PCC was 0.99 and the AUC 0.99, while in the AML dataset [45] PCC was 0.96 and the AUC 0.96. These results allow discarding a significant amount of type II error in the method.

### Dissecting pathway functionalities

#### Signaling-dependent mechanisms of colorectal cancer progression

**Figure 2:**
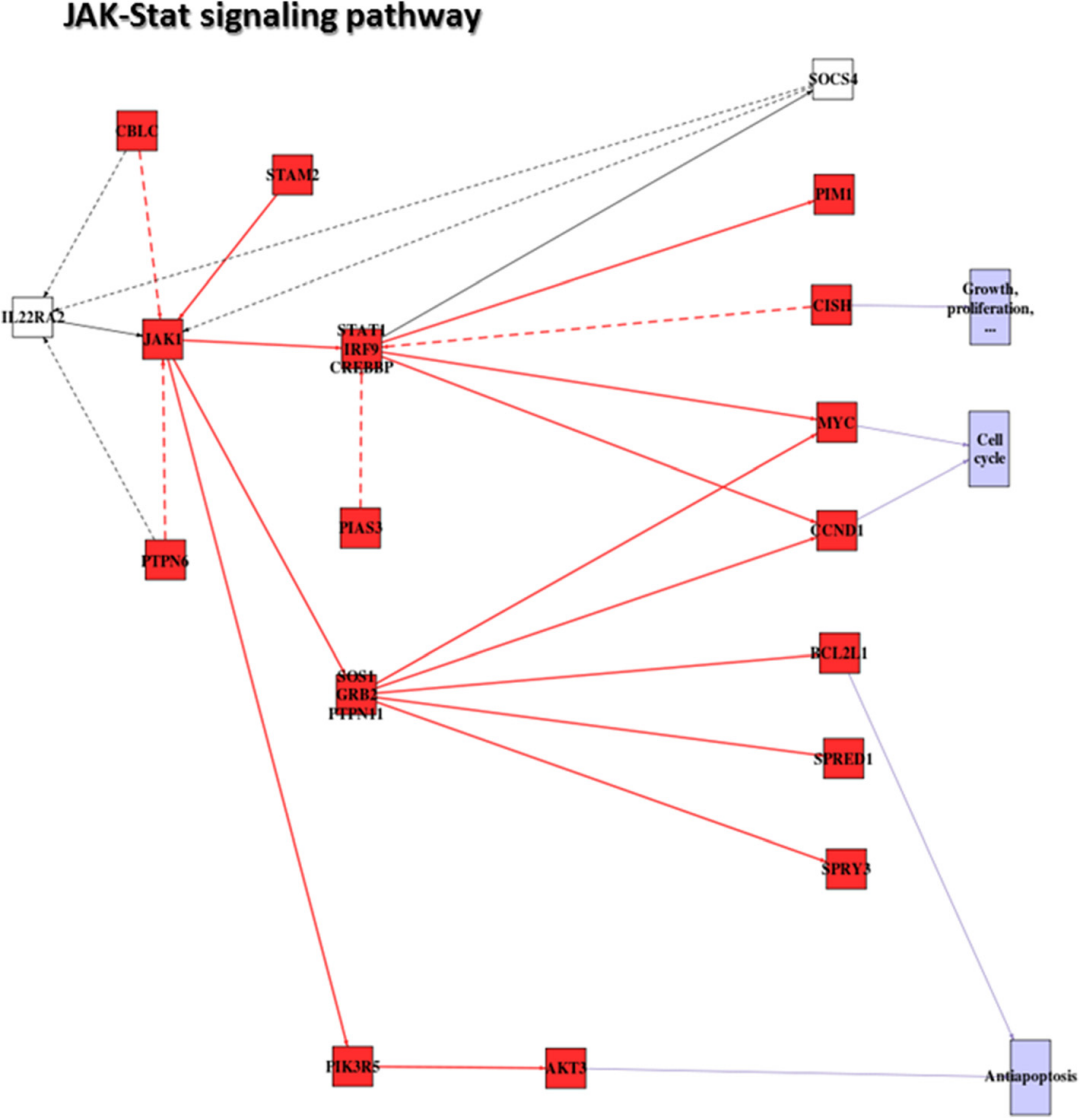
**Model of the** ***JAK-STAT*** **pathway in CRC with the corresponding significant changes in the signaling circuit activities.** Red nodes indicate activated circuits in the CRC patients with respect to the healthy controls and blue nodes indicate circuit deactivations. Solid lines indicate activations and dashed lines indicate inhibitions.

We have used an example of colorectal cancer (CRC) to illustrate this concept. A recent microarray (Affymetrix™ array HG-U133 Plus2.0) study has been used to systematically search for genes differentially expressed in early onset CRC [48]. The study involves the comparison between 12 CRC cases and 10 healthy controls (see Material and Methods for details). Several pathways are known to be affected in CRC. For example, the proposed test detects a significant increase of signaling activity in circuits leading to growth proliferation, cell cycle and anti-apoptosis in the *Jak-STAT pathway* (see Figure 2). This result is coherent with previous results, which describe how the inhibition of this pathway induces apoptosis, cell cycle arrest, and reduces tumor cell invasion in colorectal cancer cells [49,50].

Another pathway whose activity is significantly altered is the *WNT signaling pathway*. This pathway has a canonical *Wnt/β-catenin* cascade and two non-canonical pathways named *Wnt/Planar cell polarity* (Wnt/PCP) pathway and *Wnt/Ca2+* pathway, forming part of the complete *WNT signaling pathway*. The results of the test reveal a significantly increased activity for two out of the three circuits (Additional file 1: Figure S1). Genes belonging to the Wnt/PCT pathway, such as *RhoA* or *RAC* and *JNK* are known to be up-regulated in cancer [51]. This would explain the activation of the circuit leading to *JNK*, which is one of the possible responses triggered by this pathway. Other PT-based analyses also point to this pathway as a key player in CRC [30].

A remarkable example of precise detection of a signaling circuit activity occurs in the *VEGF pathway*, whose induction is known to be involved in malignant transformation [52]. Human colorectal tumors produce vascular endothelial growth factor (*VEGF*) whose expression is up-regulated in tumor cells by cyclooxygenase-2 (*COX2*), represented in the KEGG pathway in Additional file 2: Figure S2 by node *PTGS2* (prostaglandin G/H synthase and cyclooxygenase), and directly correlated to neoangiogenesis and clinical outcome [53,54]. *COX2* catalyzes the production of PGI2, and high levels of PGE2 have been reported in colorectal adenocarcinomas in comparison with adjacent normal tissue [55]. We observed that only the circuit leading to PGI2 production, via *PTGS2* (COX2), was significantly activated (Additional file 2: Figure S2A) in this pathway.

As a general rule, it is worth noticing that the patterns of circuit activities, which account for cell functionalities, cannot be easily derived from the changes in gene expression. Additional file 2: Figure S2B illustrates how different gene (with either signaling or repressing activities) up- and down-regulations suppress and compensate to each other to finally (unexpectedly) result in no change for the rest of circuits (Additional file 2: Figure S2A). Thus, genes activated or deactivated, with no effect in this pathway, are free to play a different role in another pathway. This demonstrates the usefulness of the proposed approach that provides direct information on the specific pathway activities that result in pathologic conditions, instead of just reporting gene activities out of context.

#### Study of genetic and diet-induced murine models of obesity

**Figure 3:**
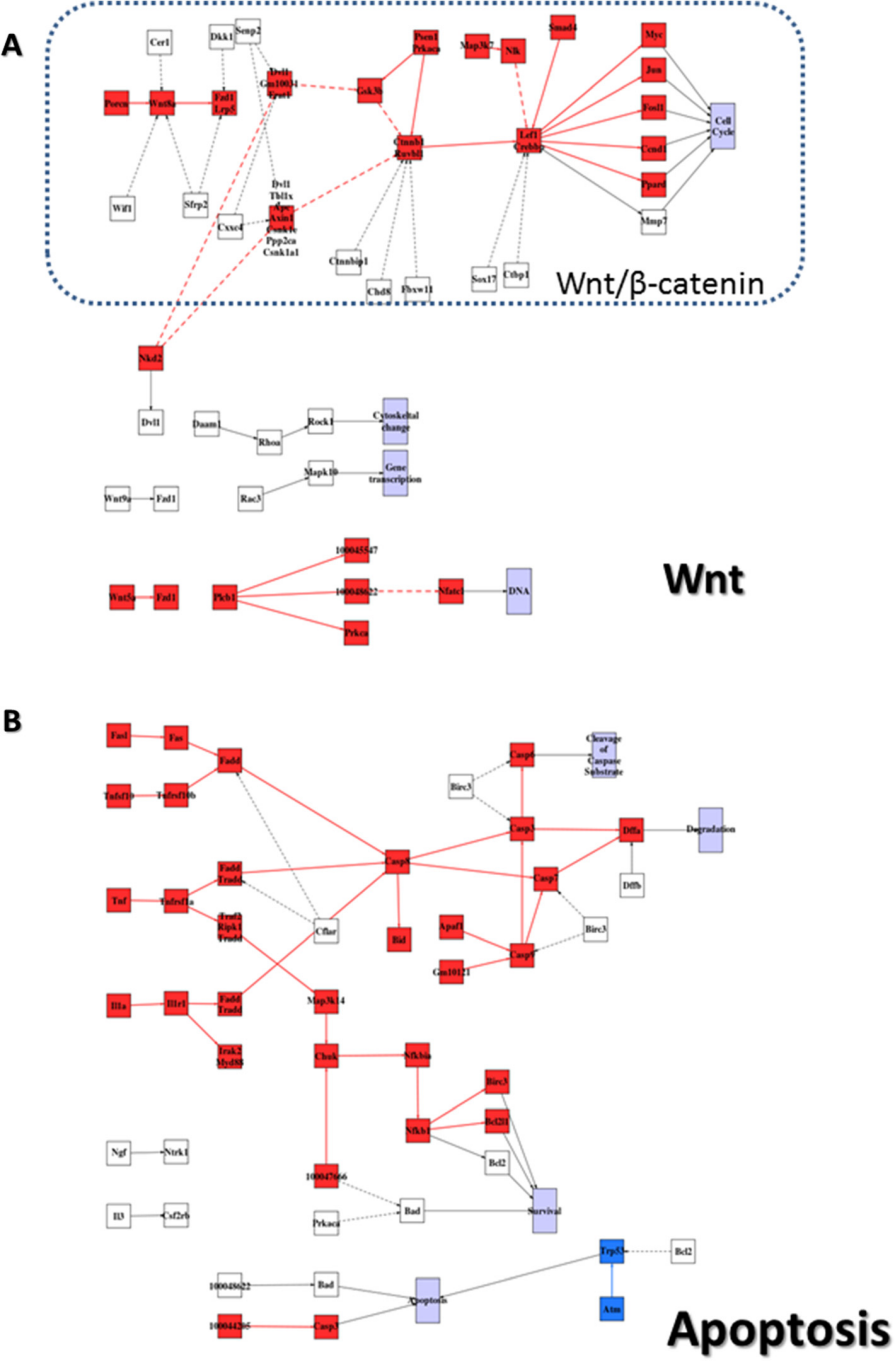
**Signaling changes in mouse models of obesity. A)** Activation within the *Wnt* pathway of circuits triggering cell cycle, in particular the Wnt/β-catenin, when wildtype mice 16 weeks old is compared to wildtype 5 weeks old, and **B)** Activation of different circuits of the *apoptosis* pathway in Ob/Ob 16 week mice when compared to the 16 week wildtype mice. Red nodes label activated circuits with respect to initial state of each comparison and blue nodes label deactivations. Solid lines indicate activations and dashed lines indicate inhibitions.

In our previous study [56], we characterized the adipose tissue macrophages (ATM) from wild type (WT) and ob/ob mice at 5 and 16 weeks of age. We showed that at 16 weeks WT ATM had a predominantly M2 antinflammatory phenotype. The M2 phenotype was also observed in 5 weeks old ob/ob ATMs. Conversely, 16 week old ob/ob ATMs had switched to a predominantly M1 proinflammatory phenotype, which was associated with severe insulin resistance, diabetes and an enrichment of proinflammatory macrophages in adipose tissue. The analysis of the activity of signaling circuits revealed specific signaling ways used to achieve the different physiological conditions. After 16 weeks, wild type animals remain able to control their carbohydrate metabolism and they are progressively expanding their adipose tissue. This process requires remodeling of adipose tissue and may be facilitated by M2 antinflammatory ATMs. Compatible with this expansion, Figure 3A documents a significant activation of circuits triggering cell cycle, in particular the Wnt/β-catenin, whose role in tissue remodeling by weight gain has already been identified [57,58]. VEGF pathway is also activated in 16w WT ATMs (Additional file 3: Figure S3). The activity of this pathway is increased when there is increased demand in adipose tissue expansion and also when there is hypoxia as part of an adaptation to increase vascularization in the tissue. In some way, increased demands for growth results in hypoxia which is compensated by increased vascularization. This result is compatible with macrophages producing VEGF and contributing to remodeling of adipose tissue when it is expanding as part of the age related growth [59]. On the other hand, Figure 3B documents the generalized activation of different circuits of the apoptosis pathway in ob/ob 16 week mice when compared to the 16 week WT mice. Degradation and Cleavage of caspase substrate is activated. However the relationship between survival and apoptosis changes with respect to the 16 week WT mice. Survival is activated and the circuits triggering apoptosis are different. These anomalous activities in apoptosis pathways in ATM macrophages from ob/ob 16 weeks are in agreement with the characteristic huge amount of fat deposition, inflammatory responses and adipocyte crowns of apoptotic adipocytes.

#### Signaling changes in human bone marrow hematopoietic stem cells

In the human hematopoietic system, aging is concomitant with decreased bone marrow cellularity and decreased adaptive immune system function. On the other hand it is also associated to increased incidence of anemia and other hematological disorders and malignancies. It has been suggest that changes within the hematopoietic stem cell (HSC) population during aging contribute significantly to the manifestation of these age-associated hematopoietic pathologies in mice [60].

**Figure 4:**
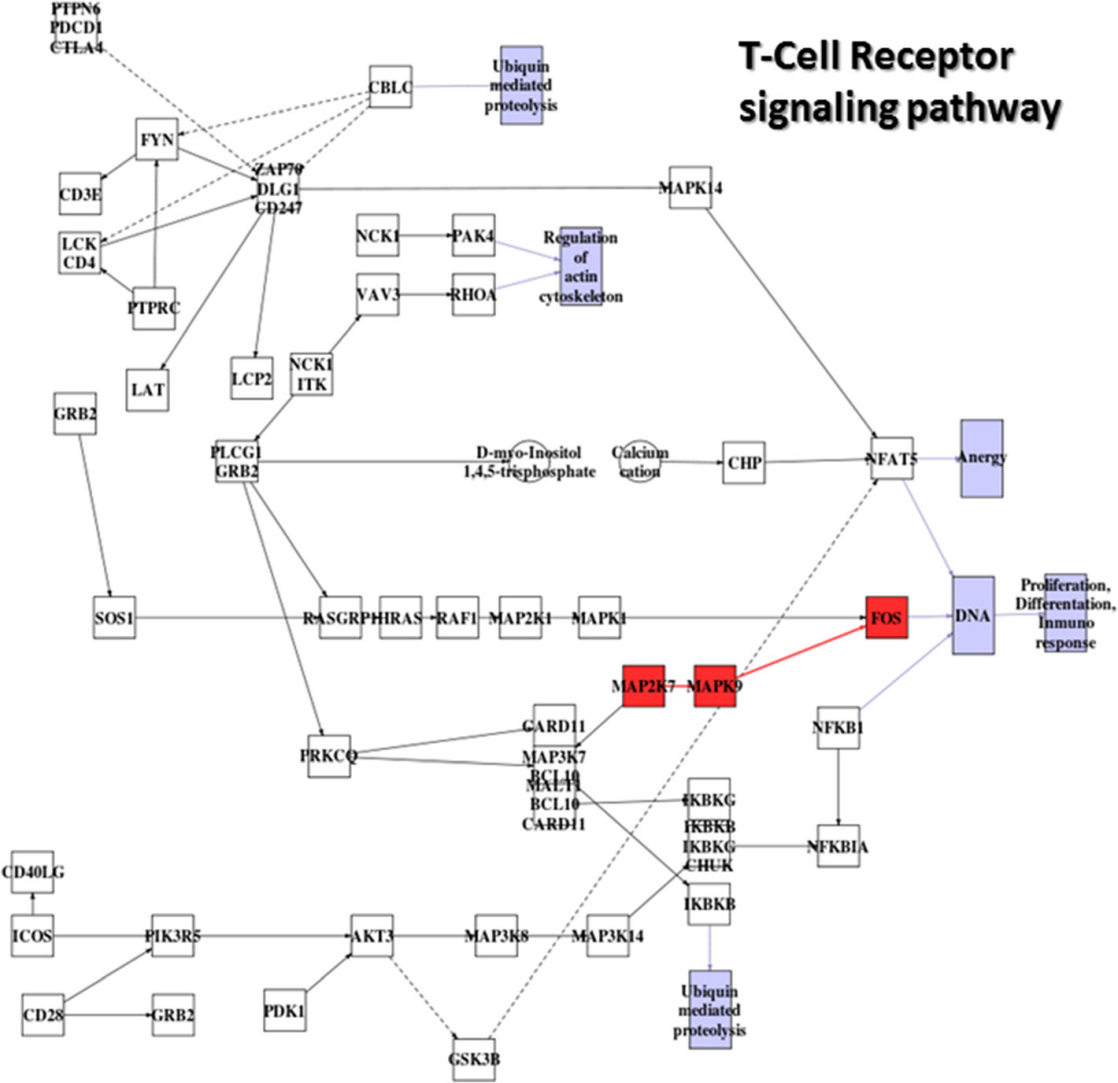
**Signaling changes in the** ***T-Cell receptor signaling*** **pathway of human bone marrow hematopoietic stem cells.** Red nodes label activated circuits with respect to initial state of each comparison and blue nodes label deactivations. Solid lines indicate activations and dashed lines indicate inhibitions.

A recent gene expression profiling study revealed that aged human HSC transcriptionally up-regulate genes related to cell cycle, myeloid lineage specification, and myeloid malignancies [61]. The study highlighted a few genes that have been implicated in hematopoietic malignances and are differentially expressed between elderly and young human HSC, being age-up-regulated: *AURKA*, *FOS*, *HOXA9*, *MYC*, *TRIM13,* while *MAFF* and *FPLT3* resulted to be age-down-regulated [61]. Our analysis of signaling circuit activity detected activation in the circuit ending in the *FOS* gene in the T-Cell receptor signaling pathway. This circuit triggers proliferation, differentiation and immune response (see Figure 4). Actually, gene set analysis carried out in the original study suggests that slight but coordinate deregulations in genes associated to *Cell cycle, Hematological System Development and Function, Cellular Growth and Proliferation* and *Humoral Inmune Response* occurred in elderly HSC when compared to young cells [61]. In other words, the original study discovered only the gene expression signatures of the processes whose activation mechanisms is reported in detail by the signaling circuit activation method proposed here.

#### Cross-talk between pathways: a case study of deregulation of programmed cell death in Fanconi Anemia

Signaling pathways are connected among them and such connections can also be detected using the method proposed here. Furthermore, the results can potentially reveal the precise mechanism by which a pathway specifically triggers a particular biological response in a second pathway. Since pathways work cooperatively, the incorrect activation of a specific circuit can activate a wrong circuit in another pathway creating a perturbed signaling cascade that could eventually result in a disease condition. The observation of the combined behavior of all the signaling pathways in a diseased cell can eventually reveal interesting details of the overall mechanism of the disease and help in the inference of ways of intervention. We illustrate this concept with the detection of cross-talk between pathways in an example of Fanconi Anemia (FA), a disease in which signaling is known to play a relevant role. FA is a rare chromosome instability syndrome characterized by aplastic anemia as well as cancer and leukemia susceptibility [62]. It has been proposed that disruption of the apoptotic control, a hallmark of FA, explains the phenotype of the disease to some extent [63]. A recent study used gene expression microarrays to identify differences at the transcription level in bone marrow cells between normal volunteers and FA patients [64]. Eleven normal volunteers and 21 patients were studied. Gene expression datasets for FA were obtained from the GEO database (see methods for details).

**Figure 5:**
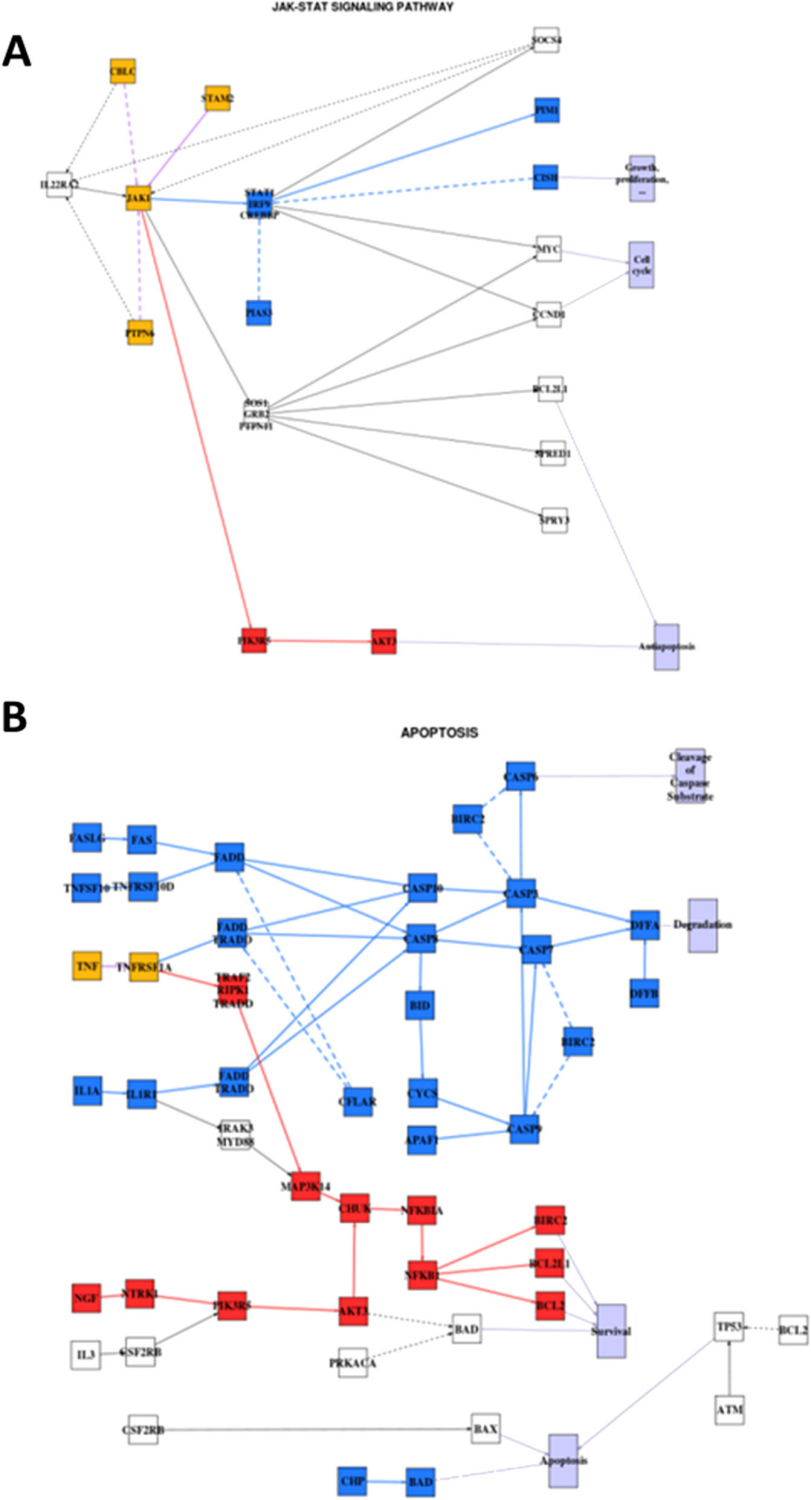
**Models of the A) JAK-STAT and B) Apoptosis pathways in FA along with the corresponding significant changes in the signaling circuit activities.** Red nodes label activated circuits in the FA patients with respect to the healthy controls and blue nodes label deactivations. Nodes that simultaneously participate in activated and deactivated pathways are labeled in yellow.

It has been proposed that one of the genes related to the disease, *FANCC*, is involved in *Jak/STAT* signaling and apoptotic signaling [62]. The model of both pathways using the data of this experiment enables the precise identification of the mechanisms by which *Jak/STAT* pathway specifically triggers one of the survival circuits of the apoptosis pathway that eventually results in the disease. Figure 5 shows the changes in signal transmission activities across all the signaling circuits when FA patients are compared to the controls. While the activity of the signaling circuit ending in *CIHS*, leading to cell proliferation, is inhibited the activity of the circuit ending in *AKT3* is significantly activated in FA (Figure 5A). This signaling circuit, according to KEGG functional annotation, specifically triggers the antiapoptotic activity in the apoptosis pathway. Figure 5B shows a detailed description of the process of antiapoptotic activity activation. Three signaling circuits are significantly inactive in FA (the ones ending in *BAD*, *DFFA* and *CASP6*) and other three circuits are significantly active (the ones ending in *BCL2L1, BIRC2* and *BCL2*). The consequences of the observed changes in the activation status of the circuits affected can easily be understood at the light of the functions they trigger (Figure 5B). Firstly, one of the circuits triggering apoptotic response (the one ending in *BAD*) is inactivated in FA. Additionally, another two circuits triggering degradation (*DFFA*) and cleavage of caspase substrate (*CASP6*), respectively, are also deactivated. On the other hand, circuits ending in *BCL2L1, BIRC2 and BCL2*, that trigger survival, are activated. Thus, the final consequence is that the antiapoptotic pathway activity in FA increases with respect to a normal cell. This does not mean that apoptosis cannot occur in FA. Actually it does [63], most probably through the circuits ending in *BAX* and/or *TP53* (see Figure 5B)*,* whose activity is not different from the activity in normal cells. This observation suggest that known features of FA, such as hypersensitivity to DNA cross-linking agents [65,66] or chromosomal instability [66] could be a consequence of the abnormal survival of cells with damaged DNA. Actually, the authors of the original study describe that some functional categories, including “negative regulation of programmed cell death” are over-represented in the FA samples. Actually, recent reports have confirmed that FA proteins participate directly in canonical signaling pathways that influence survival and self-replication of hematopoietic cells [64].

Conventional (PT)-based methods of functional enrichment, even if they take into account the relationships between the components, fail in revealing details on the molecular mechanisms that mediate the pathologic behaviors of the apoptosis in FA. And, obviously, cannot account for the cross-talk between pathways.

### Comparison with other approaches

It is difficult to provide a reasonable comparison of the procedure proposed with other procedures because they test different aspects of the functionality of the pathway. A recent revision on pathway-based methods describe the evolution of the different algorithms proposed from those that only consider membership relationships between the genes of a pathway (SEA or Functional Class Scoring -FSC-) to those, more sophisticated, that use the topology of the pathway (PT-Based) [20]. However, the ultimate property to be tested here is the behavior of the whole pathway, while our approach checks for significant differences in the probability of activation of the individual stimulus-response signaling circuits. Only very recently, a few methods that consider sub-pathways have been proposed [28-31]. The clipper approach [28] applies first a Gaussian graphical model that deconstructs the whole graph that defines the pathway into smaller sub-graphs (cliques), that are further individually tested. The cliques are genes connected through the pathway structure but not necessarily related with the actual stimulus-response biologically relevant circuits used here. For example, a clique within a stimulus-response sub-pathway can have their genes significantly up-regulated but the signal might not be transmitted if the sub-pathway is interrupted upstream or downstream. Another approach, the DEgraph [31], uses multivariate analysis to identify differential expression patterns that are coherent with a given sub-graph structure. Again, this method uses the same approach of searching for any sub-graph within the pathway, irrespective of its biological meaning. Moreover, both approaches splits nodes composed of several proteins into multiple nodes regardless of the nature of these multi-protein nodes. The dissociation of a node which is a protein complex, where all the proteins must be present to produce an active complex, into individual nodes leads to erroneous results. We used the CRC dataset [48] to compare the signaling circuit activity method proposed here to the clipper method [28], as recently implemented in the Graphite Web application [67], and the DEgraph [31] method. Additional file 4: Table S1 shows the 85 significant pathways found. A large number of pathways, such as *Cocaine addiction*, *African trypanosomiasis*, *Long-term depression*, *Salivary secretion*, and many others completely unrelated to cancer are obvious false positives. Focusing only on cancer related pathways we consider *VEGF*, *Jak-STAT*, *ERBB* and *WNT*. In *VEGF*, clipper only detect a portion of the sub-pathway leading to the production of *COX2* via *PTGS2* (prostaglandin G/H synthase and cyclooxygenase), which is detected by our approach and is known to be activated in CRC [55,68]. Clipper and DEgraph declare so many pathways as significantly activated because they detect sub-graphs, which are portions of signaling circuits of dubious biological meaning. As previously commented for some up-and down-regulations of individual genes, some sub-graphs have not impact on biological functions triggered by signaling pathways as well. And again, in many cases the use of differential expression instead of individual gene activation or deactivations might introduce errors in the role of inhibitors in the pathways.

Additionally, two almost identical approaches that define sub-pathways in a similar manner to our signaling circuits but score them in a similar manner to SPIA [22] have recently been published [29,30]. However, the use of differential expression values in the nodes instead of actual presence/absence estimations lead to incorrect assumptions on the activities of sub-pathways, as mentioned above. Moreover, they do not provide any software for carrying out the test, which critically compromises the potential usefulness of both approaches.

In an apparent conceptual downgrade, the way in which new sub-pathway based methods check its validity implies checking whether the pathways in which they found significant sup-pathways are also significant in conventional FSC or PT-based methods or not. Although probably of limited value, we produced a similar comparison. We have used the CRC example [48] for the comparison. We have analyzed the pathways to detect significant changes in signal transmission activities in the signaling circuits between the two conditions compared. Table 1 represents the number of circuits significantly activated and deactivated in cases with respect to the controls. We compare this approach to: a) a conventional ORA test [3], which require a pre-selection of genes based on individual tests for each gene [13]; b) to a FSC test (in particular a representative of the family of GSEA tests [15]) which do not require of the previously mentioned step of pre-selection of genes; c) to a PT-based approach, the SPIA [22], which takes into account the internal structure of the pathway for the calculation of a global activity index for the whole pathway.

**Table 1:** **Analysis of the activity of the pathways by means of different approaches in the case-control comparison of early onset colorectal cancer**

	**Circuits**			**SEA**				
**PATHWAY**	**Total**	**Case**	**Control**	**UP vs DOWN**	**UP vs ALL**	**DOWN vs ALL**	**GSEA**	**SPIA**
PPAR SIGNALING PATHWAY	106	3	19	DOWN	ALL	ALL	DOWN*	INH*
ERBB SIGNALING PATHWAY	139	11	2	UP	UP	DOWN	DOWN*	INH
CALCIUM SIGNALING PATHWAY	20	2	2	DOWN	UP	DOWN	UP*	ACT
NEUROACTIVE LIGAND-RECEPTOR INTERACTION	7	0	0	UP	UP	ALL	UP*	ACT
APOPTOSIS	28	0	0	DOWN	ALL	DOWN	DOWN*	INH
WNT SIGNALING PATHWAY	37	6	6	UP	UP	DOWN	DOWN*	INH
NOTCH SIGNALING PATHWAY	14	0	0	DOWN	ALL	ALL	DOWN	INH
VEGF SIGNALING PATHWAY	10	2	0	DOWN	ALL	ALL	DOWN	INH
CELL ADHESION MOLECULES	43	6	3	UP	UP	ALL	UP*	– ^1^
GAP JUNCTION	17	4	0	UP	UP	ALL	UP*	ACT
ANTIGEN PROCESSING AND PRESENTATION	6	0	0	DOWN	ALL	DOWN	UP	ACT
TOLL-LIKE RECEPTOR SIGNALING PATHWAY	103	0	0	UP	UP	DOWN	UP*	INH
JAK-STAT SIGNALING PATHWAY	7	7	0	DOWN	ALL	DOWN	UP*	INH
B CELL RECEPTOR SIGNALING PATHWAY	10	0	0	UP	UP	ALL	UP*	INH
Fc EPSILON RI SIGNALING PATHWAY	7	0	0	UP	ALL	ALL	DOWN*	INH
INSULIN SIGNALING PATHWAY	54	1	0	DOWN	UP	ALL	DOWN*	INH
GnRH SIGNALING PATHWAY	9	0	0	UP	UP	ALL	DOWN*	ACT
MELANOGENESIS	8	1	0	UP	ALL	ALL	**UP**	ACT
ADIPOCYTOKINE SIGNALING PATHWAY	31	0	2	DOWN	ALL	ALL	UP	INH

1 – This pathway is not implemented in SPIA.

The first column contains the name of the pathway. The next three columns, collectively labeled as circuits, list the number of sub-pathways in the pathways (**Total**) and the number of them significantly activated in cases with respect to controls (**Case**) or vice versa (**Control**) in the comparison, respectively. The three next columns, collectively labeled as **SEA**, list the results of a conventional functional enrichment test [3] in three situations: **UP vs DOWN**) when the significantly upregulated genes are compared to the significantly downregulated genes, **UP vs ALL**) when significantly upregulated genes are compared to rest of genes, and **DOWN vs ALL**) when significantly downregulated genes are compared to the rest of genes. UP, DOWN and ALL means where the major part of the pathway lies in the comparison. Significantly up- and downregulated genes are obtained by a conventional t-test with multiple test adjustment as implemented in the Babelomics program [89]. Although the trends of the results are coincident with the other analyses, none of them resulted significant. The column labeled **GSEA** contains a version of GSEA test [15] implemented in the Babelomics program. The * and the boldface indicate the trend is significant according to the test. The last column, labeled as **SPIA**, contains the result of the application of the pathway impact analysis [23].

No main discrepancies were observed among the significant values reported by the SPIA method and the proposed method. In some cases, the behavior of SPIA is closer to the GSEA, probably because both methods return a global pathway score (despite the fact that SPIA weights take into account the pathway structure, thus providing a more accurate description of the whole behavior of the pathway.) However, our method is testing a different aspect of the activity of a pathway than SPIA and GSEA do. For example, a pathway known to be active in cancer is the VEGF signaling pathway, whose inhibition has been suggested as an anticancer therapy [69]. Several circuits in this pathway were detected as activated by the proposed approach but not by the other approaches (see Table 1). Likewise, the relationship of JAK-STAT signaling pathway to colorectal cancer is known, given that its disruption reduces tumor cell invasion in this cancer [49]. Again, circuits within the JAK-STAT pathway were found activated by the approach proposed here and by GSEA but not by SPIA. The apparent superior sensitivity of GSEA methodologies in this case is probably due to its lower specificity [70]. The general conclusion derived from the comparison is that the method proposed here finds circuits activated in pathways which are detected as significant by whole-pathway FSC or PT-based methods. As expected, our more sensitive method also detects activations of specific signaling circuits in pathways which were non-significant in FSC tests.

## Conclusions

Because of the complexity of the cell, functional traits cannot be understood as the result of the action of only one or a few genes [71,72]. The multigenic (and non-necessarily additive) nature of many common phenotypes (including common diseases) is one of the causes of the low statistical power rendered by conventional marker- or gene-based testing strategies when applied to genomic data (e.g. genotyping or transcriptomic) [73,74]. Specific strategies for pathway analysis (such as SEA, FSC and PT-based methods that test the collective activity of sets of functionally related genes as defined in GO, KEGG, etc.), despite more sensitive than gene-based approaches, still provide a very poor detail on the actual functionalities affected in the cell.

Therefore, in a realistic scenario, the conventional paradigm that link disease to the failure of a particular gene needs to be changed for a systems-biology-oriented view [75-77], in which the causative factor of the disease is not the gene itself but perturbations of the combined activity of several genes, functionally related through pathways (the signaling circuit here). Thus, the anomalous activity of one or several circuits, responsible for specific cell functionalities, could be the ultimate cause of the disease. Probabilistic models have been used some time ago in theoretical approaches to understand regulatory networks [78-80] and, more recently, for different types of studies that involved the characterization of regulatory or signaling circuits [37-40,81]. Increasing the knowledge on the way in which signaling circuits operate is crucial: changes in the activity of the biological functions triggered by signaling pathways (via the circuits that compose them) are behind the mechanisms of several diseases. Here, we extend the idea of probabilistic model to the study of the functional consequences of gene expression in the context of signaling pathways. Simple but efficient probabilistic models of pathway activity have been developed here for a collection of signaling pathways from KEGG [10].

Unlike other conventional approaches, where either the activity of individual genes or the activity of the complete pathway was tested, this approach dissects the structure of the pathway and tests the activity of the stimulus-response signaling circuits. Actually, the notion of pathway is somehow an arbitrary concept based, in some cases, on historical or theoretical concepts that may include many different actual functional roles. The aim of the method proposed here is focusing on the actual elementary functionalities triggered by these circuits within the context of the pathway, whose failure (wrong activation state, tested by the method) can be associated to the disease. There are only a few recently published methods that consider sub-pathways, however they present some problems that seriously limit its applicability and compromises the accuracy of the results provided [28-31]. Firstly, some of them do not consider biologically meaningful sub-pathways, such as the signaling circuits considered here, but rather any sequence of connected proteins (sub-graphs), no mater of their relevance in the signaling transmission process [28,31]. This produces false positives because pathways are declared as significantly activated when some of such topologically-defined sub-graphs are active, regardless of their contribution to effective signal transmission. Even if the circuits are reasonably well defined [29,30], many nodes, often composed by several proteins, are not well resolved, which results in erroneous estimations of the states of the circuits. Moreover, all these methods use differential expression values in the nodes instead of actual probabilities of node activity, which lead to incorrect assumptions on the activities of sub-pathways in general and signaling circuits in particular. In addition some of the methods do not provide any software, which critically compromises their potential use [29,30].

The analysis of type I and type II errors demonstrated the accuracy of the methodology proposed. The examples presented in this paper illustrate the use of the concept of the probability of stimulus-response circuit integrity for signal transmission in different scenarios including cross-talk between pathways.

We have focused on gene expression data obtained from microarrays purely by practical reasons of availability: it is the most abundant genomic data publicly available. Obviously, gene expression values can be obtained by other methodologies, providing the data compared are in the same scale (this is the objective of the normalization process). In particular, it is likely that in a near future most of the gene expression measurements will be obtained from RNA-seq experiments [82]. Different measurements for differential gene expression have been proposed, that can accurately account for the estimation of the differential gene expression [83] that could be easily adapted to measure the differential activation of a signaling circuit.

Detailed pathway models helps to understand the disease mechanism [72] as well as throw light on possible mechanisms of drug action [84]. Such models facilitate the advent of a real systems medicine where interventions over particular molecules can be understood within a systems context [76,77]. Actually, the method proposed fosters the conceptual transition from gene-based biomarkers to mechanism-based biomarkers (the signaling circuits). These can be derived from the gene expression values combined in a way that have a biological meaning and potentially account for the disease mechanism. Moreover, pathway models will likely play an important role in the interpretation of different types of genomic data apart from gene expression. Thus, models of pathways can also be used to study the possible impact of mutations found in exome or whole genome resequencing experiments in the context of pathway functionality. The study of the combined effect of these mutations on the different signaling circuits and consequently the resulting cellular functionality damaged will help to understand the mechanism of the disease and to propose ways of intervention. Similarly, it will also be possible to understand the functional implications at pathway level of genome-wide regulatory constraints found in methyl-seq [85] experiments and its corresponding impact in diseases. Steps toward model the functionality of the cell will render more detailed insights into the knowledge of the living systems and will provide more powerful statistical tools to study the behavior of the cell in a diseased condition. Models developed here for the relatively simple scenario of signaling pathways really capture the network of relationships among proteins that account for particular aspects of the functionality of the cell.

## Methods

### Data sources and preprocessing

All data used in this study was downloaded from the Gene Expression Omnibus (GEO), public repository of the NCBI [86]. By the time this study was carried out, there were 169 GEO series containing microarray data generated using the Affymetrix GeneChip HG-U133 Plus 2.0 Array (GPL570 platform in the GEO data base). Only for 74 of those series raw data (Affymetrix .CEL files) were available, comprising a total of 3034 array hybridized to all kind of human samples. The same search was performed for the platforms HG-U133A and MoGene-1_0-st-v1, rendering a total of 5293 and 683 arrays, respectively. A collection of reference arrays was made with them. The raw data (.CEL files) for these platforms were downloaded. The arrays were normalized in batches of size 100 (because of memory size limitations) using the function RMA in the *affy* library [87] of Bioconductor and finally all batches were rescaled together using the “quantile” method of the *limma* library [88] of Bioconductor, implemented in the Babelomics [89] platform. Details of the procedure followed have been published elsewhere [17]. The data collection covered an ample spectrum of biological conditions including different tissues, and diseases, male and female individuals as well as cell lines.

Gene expression data for the CRC analysis were retrieved from the GEO (GSE4107). Gene expression datasets for the Fanconi Anemia analysis were obtained from GEO (GSE16334) (Affymetrix HG-U133A Array). Gene expression datasets for the murine models of obesity were downloaded from GEO (GSE36669). Human bone marrow hematopoietic stem cells gene expression data were retrieved from GEO (GSE32719). Gene expression data of pediatric acute myeloid leukemia (AML) used in the validation of the methodology were retrieved from GEO (GSE17855). Gene expression data for breast cancer and data from the study of genes with increased expression in AML were also retrieved from GEO (GSE27562 and GSE9476, respectively).

The original GEO normalization of each dataset was used. Differential gene expression control versus case samples were carried out using the Limma library [88] from Bioconductor.

### Models of KEGG's pathways

**Figure 6:**
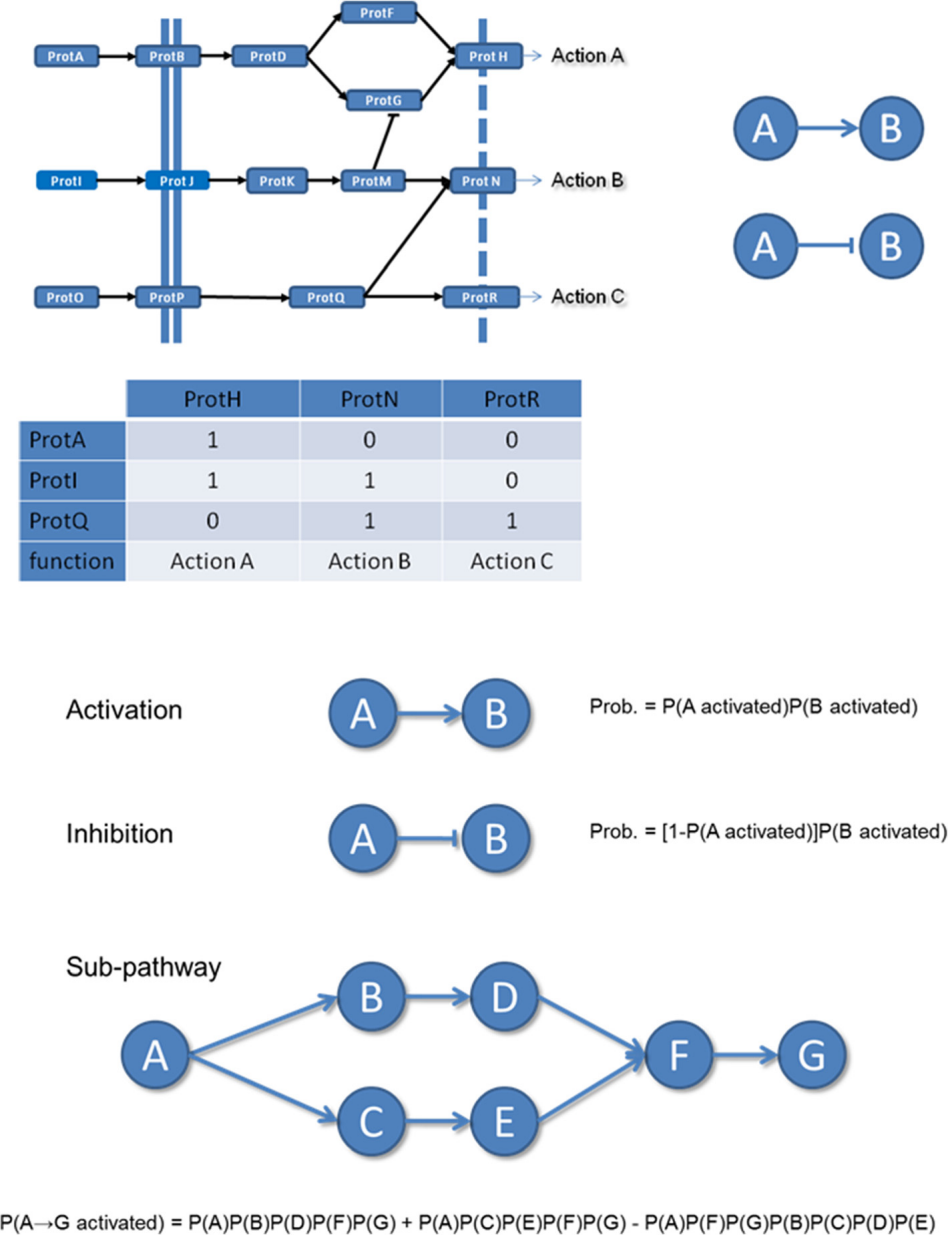
**Example pathway with three possible inputs and three possible outputs.** Any of the output proteins trigger a different functionality of the pathway. The connection matrix shows how input and output can be connected through 5 different sub-pathways. Any sub-pathway can be traversed by different paths. For example, the sub-pathway connecting ProtA to ProtH can be traversed by two different paths, represented by two sequences of nodes (that, for the sake of the simplicity are here equivalent to proteins): ProtA, ProtB, ProtD, ProtF, ProtH or ProtA, ProtB, ProtD, ProtG, ProtH. On the right, the two interactions among proteins: top right represents activation, with an arrowhead line, and bottom right represents repression with a line with no arrow. In the lower part there is an example to illustrate the way in which the probability of activation of a sub-pathway can be calculated from the combined activation of the corresponding nodes. From top to bottom: i) Probability of the transmission of the signal through an activation action, ii) Probability of the transmission of the signal through a repression action, iii) An example of a simple bifurcating sub-pathway and iv) the probability of signal transmission along this pathway.

Signaling pathways offer an excellent framework for deriving models of biological activity because their functionality can be easily conceptualized: it simply consists on the potential of transmitting a signal from the protein that receives a stimulus (signal input) to the effector protein that triggers the action (signal output) in response to the stimulus. In practical terms, any node with no incoming interactions is defined as signal input node and any node with outgoing interaction is defined as a signal output node. Then, a sub-pathway is defined as a signal entry point and the collection of intermediate connecting nodes that links it to a signal output node. We will name circuits to these particular sub-pathways in order to distinguish them from any other possible sub-pathway that does not connect signal input to signal output nodes within the pathway. Thus, we recode each pathway into a collection of circuits. Any of these circuits represent a potential elementary functionality triggered by the corresponding signal output node. Some signal output nodes have specific annotations, included in the KEGG pathway definition, about the functionality triggered, although a number of them lack annotation. Figure 6 schematizes an example with three possible inputs and three possible outputs that trigger three possible functionalities of the pathway. The connection matrix shows how input and output can be connected through 5 different circuits (non-zero entries). Circuits can have more than one sub-pathway (linear path) that connects the input to the output node. For example, Figure 6 shows how the circuit that connects ProtA to ProtH can be traversed by two different ways, represented by the following sequences of nodes: ProtA, ProtB, ProtD, ProtF, ProtH or ProtA, ProtB, ProtD, ProtG, ProtH. A node can be one individual protein, several alternative proteins or a complex of proteins.

A total of 27 KEGG pathways for *Homo sapiens* and 18 for *Mus musculus* belonging to the general categories Environmental Information Processing and Cellular Processes, which include important processes and systems such as Signal Transduction (*ERBB*, *WNT*, *NOTCH*, *JAK-STAT*, calcium, *VEGF*, *HEDGEHOG* and *mTOR* signaling pathways), Signaling Molecules and Interaction (*neuroactive ligand-receptor interaction, cell adhesion molecules, cytokine-cytokine receptor interaction* and *EMC-receptor interaction*), Cell Growth and Death (*apoptosis and p53 signaling pathway*), Cell Communication (*GAP junction* and *tight junction*), Endocrine System *(insulin signaling pathway, adipocytokine signaling pathway, PPAR signaling pathway, GnRH signaling pathway* and *melanogenesis*) and Immune System *(toll-like receptor signaling pathway, B cell receptor signaling pathway, T cell receptor signaling pathway, Fc epsilon RI signaling pathway, antigen processing and presentation,* and *chemokine signaling pathway*), could be coded in this way.

The information about the topology of each pathway was extracted from the corresponding KEGG’s XML files. The KEGG database provides information on the genes and their corresponding relationships, making it possible to reconstruct each pathway from its corresponding XML-formatted file (in KGML format - KEGG Markup Language). The Dijkstra's algorithm [90] is used to discover all the possible sub-pathways between an input and an output node.

Only two types of relationships are considered: activations and inhibitions. These are coded in the XML file, either directly or through a more detailed description of the mechanism by which the molecules perform the action. Thus, according to KEGG nomenclature, the labels *Phosphorylation*, *Dephosphorylation*, *Indirect*, *Expression* and *Compound* are initially considered activations, unless the contrary is specified. That is, if a relationship is labeled as *phosphorylation,* we consider that the signal is transmitted to the next node, however, if it is labeled as both, *phosphorylation* and *inhibition*, we consider that the relationship is a *phosphorylation* that is inhibiting the activity of the next node in the pathway. On the other hand, *Ubiquitination*, and *Inhibition* are considered inhibitions. When several proteins are linked by binding/association relationships, or are indicated as a group in the XML file, they are considered as a single node that need to be simultaneously activated to transmit the signal.

Once a pathway is represented in this way, the probabilities of signal transmission from any entry point (stimulus receptor) in the input layer to any exit point (protein that triggers the response to the stimulus) in the output layer can be calculated, providing the activation state of the nodes connecting them are known. Moreover, changes in signal transmission probabilities when comparing different experimental situations can be tested.

### Estimation of the activation state of a node in the pathway

As mentioned above, a node can be composed of only one protein, several alternative proteins or a complex of proteins. The probability of activity in any of these scenarios can be conceptually derived from the probability of activation of a protein. Commonly, the presence of the transcript(s) corresponding to a gene is taken as a proxy of the presence of the resultant gene product [26]. We assume that two alternative states are possible for a gene: active and inactive. The activation state can be inferred from the expression value observed across many samples. It has been previously described that gene expression values for the inactive state often follow an exponential distribution, while the values corresponding to the activated state tend to follow a normal distribution [26]. Microarrays provide indirect measurements of the gene expression values via sets of probes (namely probesets in the Affymetrix™ technology). Following this, we have calculated the mixture of distributions that best reflects the level of expression of any gene (represented by the corresponding probeset in the microarray) across the collection of microarrays of reference described above. The Expectation-Maximization algorithm, as implemented in the R package *mixdist* [91], was used to fit the expression data to a mixture of two distributions (that can be normal or gamma, which are generalizations of the distributions proposed for this scenario [26,92]). For any probeset for each gene, the program calculates the two highest peaks in the distribution of expression values, and uses them as starting point for the calculation of the mixtures. Then, it calculates the distribution that best suits the data, the proportion in the population of each component of the mixture and the associated parameters to each distribution which are calculated from its mean and standard deviation.

Once such calculations are available for any of the probesets across all the microarrays studied, the distributions obtained can be used to estimate the probability of activation for this probeset in a different microarray [26,92,93].

The probability of a probe being activated given its expression level x can be calculated from the Bayes’ Theorem:

$$ P\left( Active\Big|x\right)=\frac{p\left(x\Big| Active\right)P(Active)}{p(x)} $$

where, according to the Law of total probability:

$$ p(x)=p\left(x\Big| Active\right)P(Active)+p\left(x\Big| Inactive\right)P(Inactive) $$

That, in terms of our mixtures can be written as:

$$ p\left( Activated\Big|x\right)=\frac{\pi_1{p}_1(x)}{\pi_0{p}_0(x)+{\pi}_1{p}_1(x)} $$

Where π_0_ = *P*(*Inactive*) and π_1_ = *P*(*Active*) are the proportions in the population for each distribution, that are calculated by the mixdist package, and *p*_*0*_(*x*) = *p*(*x*|*Inactive*) and p_1_(*x*) = p(*x*|*Active*) are the distributions that form the mixture.

Although the distributions derived from these particular datasets are only applicable to the particular microarray platforms used in this study (HG-U133 Plus 2.0, HG-133A and MoGene-1_0-st-v1), it is straightforward to produce similar distributions for the corresponding probesets of reference in other microarray platforms (actually, more platforms are available in the implementation of the method). Probesets mapping on multiple genes are discarded from the calculations.

Given a microarray, the probabilities for each probeset of being activated can be used to derive the probabilities of gene activation (proxies for the corresponding protein activation probabilities) in the experiment studied. We use the 90% percentile of the distribution of the probeset activation probabilities as the value of probability of gene activation. The rationale behind this approach is to take the highest signal of hybridization of a probeset as an evidence of the existence of gene activity trying to avoid the most extreme values. In this way we choose a representative value for each gene [94] by means of which we minimize the false positives and negatives derived either from outliers or (more frequently) from unsuccessful probe hybridizations.

Nodes in the pathway are composed of one or more proteins. In the case of nodes of multiple proteins, these can be independent or can be part of a protein complex. When the node is composed by a single protein, the probability of node activity is the probability of activation of this protein. The second scenario corresponds to a node composed by more than one alternative protein. These are supposed to be redundant in its activity, meaning that the existence of only one of these proteins would be enough for the transmission of the signal. In this case, all the probes corresponding to all the genes in the node are taken together and the 90% percentile of the distribution of their activation probabilities is taken as the value of probability of node activation. The third scenario, corresponding to a complex of proteins, is slightly different because all the proteins are simultaneously necessary for its integrity. Unlike in the previous case, we consider that the activity of the node depends on the simultaneous presence of all their components. Consequently, we consider that the probability of having this type of node active is conditioned by the lowest probability of having any of the proteins of the complex active. The lowest of these probabilities will be the limiting factor for the integrity (and consequently, the activity) of the node. Of course, the scenarios for the nodes are idealizations of a reality which may be much more complex, but they have demonstrated to represent a realistic enough scenario and to work in practical terms [95].

### Probability of signal transmission along a circuit

As stated above, a signaling circuit is defined by an input node (the protein that receives the stimulus), an output node (the protein that triggers the response to the stimulus) and all the intermediate nodes connecting them. Thus, the probability of signal transmission along a circuit can be derived from the probability of **a)** having all the nodes connecting the input node to the output node active and **b)** having all the nodes that are inhibitors of nodes in the pathway in an activation state compatible with the transmission of the signal. When there are several possible ways (bi- or multi-furcations) to transmit the signal from the input node to the output node (linear sub-pathways), the formula of the probability of the union of several events can be used. Thus, once input and output nodes and the topology of the intermediate nodes has been defined for a particular circuit, and the probabilities of activation for each node in the pathway has been estimated (as described in the previous section), the probability of signal transmission across any circuit can be calculated as:

$$ P\left({\cup}_{k=1}^n{A}_k\right)={\displaystyle \sum_{k=1}^n}P\ \left({A}_k\right)-{\displaystyle \sum_{i<j}}P\ \left({A}_i\ {\displaystyle \cap }{A}_j\right)+{\displaystyle \sum_{i<j<k}}P\ \left({A}_i\ {\displaystyle \cap}\kern0.1em {A}_j{\displaystyle \cap}\kern0.1em {A}_k\right) + \dots +{\left(-1\right)}^{n+1}\ P\left({\cup}_{k=1}^n{A}_k\right) $$

being *n* the number of linear paths conforming a circuit and being *A*_*i*_ (*i* = 1,…,*n*) any of the paths in the circuit. Thus, the resulting probability, which can be assimilated to the probability of signal transmission across the circuit, is estimated as the probability of the union of all the linear paths that form this circuit.

Figure 6 (lower part) illustrates with a very simple example the way in which the probability of combined activation can be used to calculate the probability of activation (signal transmission) of a circuit.

Circuits containing loops cannot be modeled under this simple approach and are not considered here. Specifically, this situation occurs in four pathways where we eliminated the following number of circuits (hsa04150: mTOR signaling pathway, hsa04612: Antigen processing and presentation, hsa04630: Jak-STAT signaling pathway and hsa04920: Adipocytokine signaling pathway, in which 11, 3, 18 and 22 loops were removed, respectively).

### Comparing pathways and signal transmission in two experimental conditions

Since we are using gene expression values as proxies of protein activation statuses, the resulting probabilities of signal transmission across circuits at mRNA level might not have an exact correspondence with the protein level. In other words, the value of probability obtained for a circuit could not be by itself very informative about the real activation status of the circuit. However, the comparison of two conditions will render results with a clear biological interpretation: a significant change in the probability of signal transmission along a circuit is most probably accounting for a real change it the cellular response to any stimulus. In that case it can be anticipated that changes in mRNA levels causing significant changes in signal transmission circuit status will most likely be accompanied by the corresponding change at protein levels.

This method seeks to assess the activation probability of every circuit, representing a canonical functionality within the pathway, given the status of the sample (e.g., disease, control, etc.) In order to achieve that, we use the Wilcoxon test [96] (implemented in R as the *wilcox.test* function) to compare the difference of activation probability of each individual circuit in the pathway. Then we use a False Discovery Rate (FDR) [97] control to correct the p-values obtained for each circuit in the pathway. This p-value can be used to detect circuits with a significant difference of activation between conditions. The *wilcox.test* function returns the location parameter that indicates which is the activated condition when a circuit was found to be significantly activated. Depending on the topology of the pathway a few genes can produce a remarkable change in the number of circuits activated/deactivated or vice versa.

## Methods for functional enrichment

In order to compare the proposed approach to other pathway-based methods we have used different algorithms for functional analysis, namely singular enrichment analysis (SEA), gene set enrichment analysis (GSEA), and impact analysis [23]. The FatiGO [3] is a widely used SEA implementation, which is included in the Babelomics web-based package [89]. For the GSEA we have used another implementation developed by us [15], also included in the Babelomics package [89]. For the SPIA we have used the program provided by the authors [23].

## Sample classification using circuit activation statuses as features for sensitivity assessment

In order to test the sensitivity of the method for the estimation of the probabilities of circuit activation, we have used them as features to predict disease class. A low misclassification rate can be considered an appropriate proxy for a low type II error rate [26,35]. We have used Support Vector Machine (SVM) [46] for the classification of the samples. The accuracy of the classification obtained was evaluated by ten-fold cross validation [47], using the following parameters: proportion of correct classification (PCC) and the area under the curve (AUC).

## Representation of the results

A web interface that implements the test described above has been developed [98] and is available at: http://pathiways.babelomics.org. The results are displayed in a table that contains each circuit and its corresponding p-value, its FDR corrected p-value and its estimation of the location parameter. A graphical representation of the results in the pathway context of a KEGG-like map is also provided. This representation is obtained using the R package *igraph* [99]. Nodes belonging to circuits significantly more activated in the first condition appear in blue while nodes significantly more activated in the second condition appear in red. Nodes belonging to several circuits with different activation statuses appear in yellow. Activations are represented by solid arrows and inhibitions by dashed arrows.

## Additional files

Additional file 1: Figure S1. Model of the *Wnt signaling* pathway in CRC with the corresponding significant changes in the signaling circuit activities. Red nodes indicate activated circuits in the CRC patients with respect to the healthy controls and blue nodes indicate circuit deactivations. Additional file 2: Figure S2. Model of the VEGF signaling pathway in CRC with: A) the corresponding significant changes in the signaling circuit activities. Red nodes indicate activated circuits in the CRC patients with respect to the healthy controls and blue nodes indicate circuit deactivations. B) Individual differential gene expression values in the nodes of the same pathway. Red nodes indicate genes over-expressed in CRC with respect to controls and blue nodes the opposite situation. Additional file 3: Figure S3. Signaling changes in mouse models of obesity. Activation of circuits within the *VEGF* pathway in wildtype mice 16 weeks old. Red nodes label activated circuits with respect to initial state of each comparison and blue nodes label deactivations. Additional file 4: Table S1. Significant pathways obtained upon the application of the clipper method on KEGG pathways as implemented in the Graphite Web program to the CRC dataset.

## Abbreviations

ATM: Adipose tissue macrophages
CRC: Colorectal cancer
FA: Fanconi anemia
FDR: False discovery rate
FSC: Functional class scoring
GEO: Gene expression omnibus
GO: Gene ontology
GSEA: Gene set enrichment analysis
HSC: Hematopoietic stem cell
KEGG: Kioto encyclopedia of genes and genomes
SEA: Single enrichment analysis
SPIA: Signaling pathway impact analysis
WT: Wild type
