# Supplementary figures and images for: Understanding disease mechanisms with models of signaling pathway activities
Source: BMC Syst Biol. 2014 Oct 25;8:121. doi: 10.1186/s12918-014-0121-3 (PMC4213475; doi:10.1186/s12918-014-0121-3)

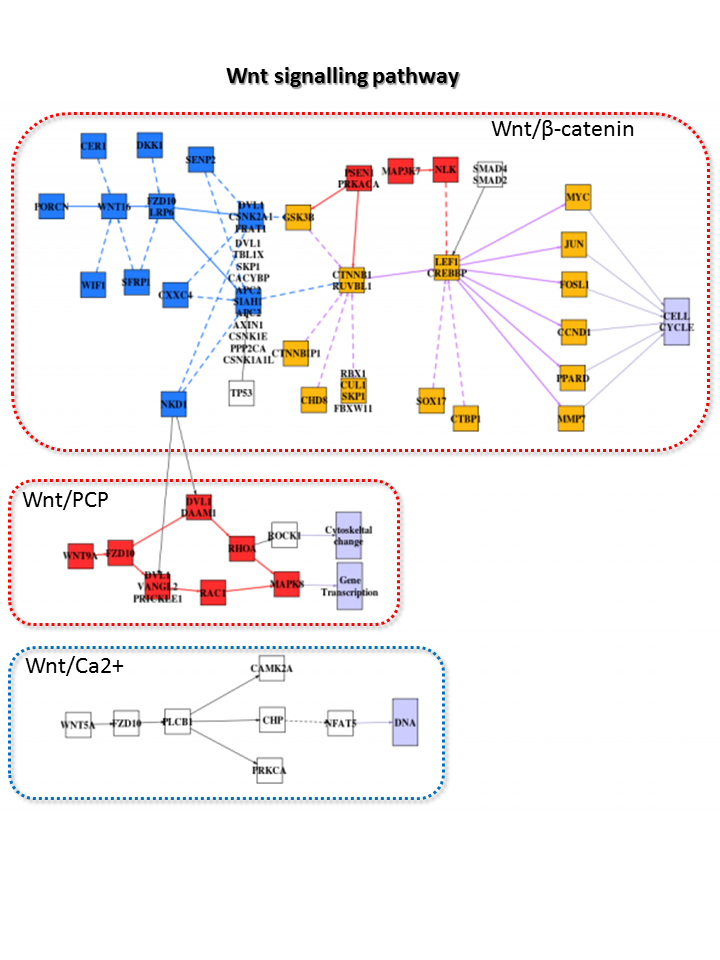

Supplement: Additional file 1: Figure S1. — Model of the Wnt signaling pathway in CRC with the corresponding significant changes in the signaling circuit activities. Red nodes indicate activated circuits in the CRC patients with respect to the healthy controls and blue nodes indicate circuit deactivations. [file 12918_2014_121_MOESM1_ESM.tiff]

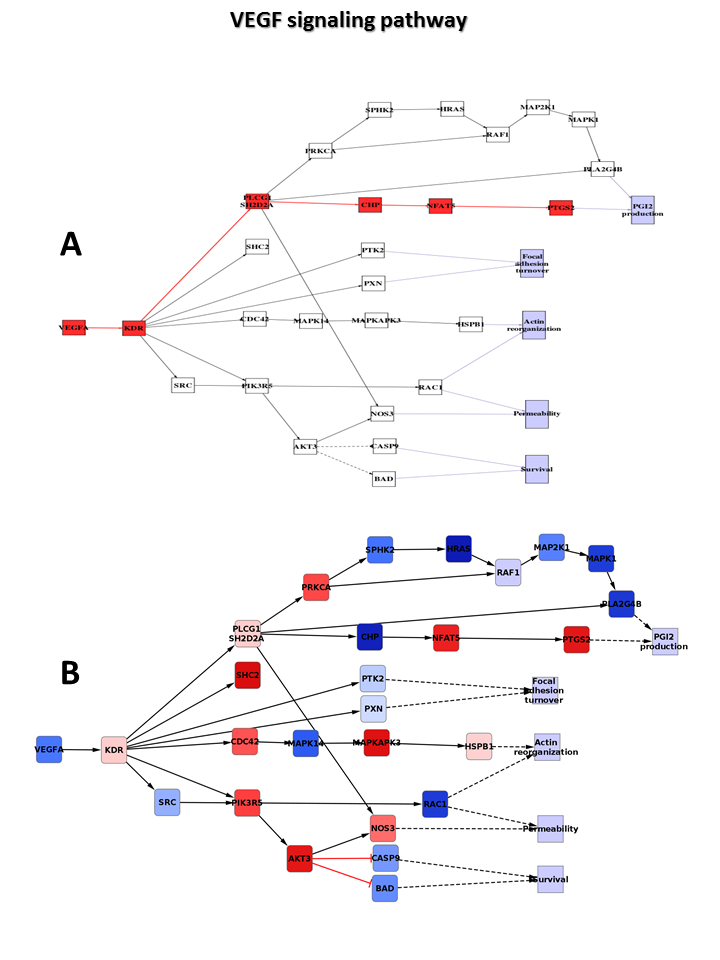

Supplement: Additional file 2: Figure S2. — Model of the VEGF signaling pathway in CRC with: A) the corresponding significant changes in the signaling circuit activities. Red nodes indicate activated circuits in the CRC patients with respect to the healthy controls and blue nodes indicate circuit deactivations. B) Individual differential gene expression values in the nodes of the same pathway. Red nodes indicate genes over-expressed in CRC with respect to controls and blue nodes the opposite situation. [file 12918_2014_121_MOESM2_ESM.tiff]

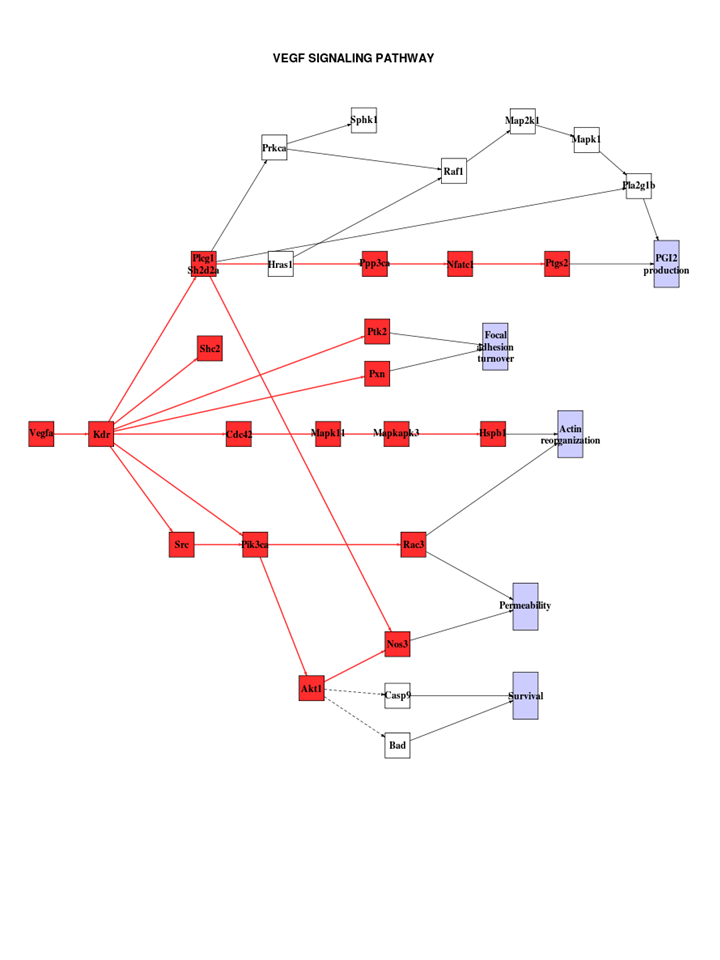

Supplement: Additional file 3: Figure S3. — Signaling changes in mouse models of obesity. Activation of circuits within the VEGF pathway in wildtype mice 16 weeks old. Red nodes label activated circuits with respect to initial state of each comparison and blue nodes label deactivations. [file 12918_2014_121_MOESM3_ESM.tiff]
