# Supplementary material for: Understanding disease mechanisms with models of signaling pathway activities
Source: BMC Syst Biol. 2014 Oct 25;8:121. doi: 10.1186/s12918-014-0121-3 (PMC4213475; doi:10.1186/s12918-014-0121-3)
Supplement: Additional file 4: Table S1. — Significant pathways obtained upon the application of the clipper method on KEGG pathways as implemented in the Graphite Web program to the CRC dataset. [file 12918_2014_121_MOESM4_ESM.docx]

**Additional file 1: Table S1**. Significant pathways obtained upon the application of the clipper method on KEGG pathways as implemented in the Graphite Web program to the CRC dataset.

| Pathway | alphaMean | alphaVar |
| --- | --- | --- |
| Adherens junction | 0 | 0 |
| Measles | 0 | 0 |
| Toll-like receptor signaling pathway | 0 | 0 |
| Vascular smooth muscle contraction | 0 | 0 |
| Viral myocarditis | 0 | 0 |
| Acute myeloid leukemia | 0 | 0.01 |
| Glutamatergic synapse | 0.01 | 0 |
| Pathogenic Escherichia coli infection | 0 | 0.01 |
| Prostate cancer | 0 | 0.01 |
| Circadian entrainment | 0 | 0.02 |
| Dopaminergic synapse | 0.01 | 0.01 |
| Epstein-Barr virus infection | 0 | 0.02 |
| Salivary secretion | 0.01 | 0.01 |
| Pancreatic secretion | 0 | 0.03 |
| Cell adhesion molecules (CAMs) | 0.05 | 0 |
| Chronic myeloid leukemia | 0 | 0.05 |
| Gap junction | 0 | 0.05 |
| Hepatitis B | 0.01 | 0.04 |
| Shigellosis | 0.05 | 0 |
| Jak-STAT signaling pathway | 0 | 0.06 |
| Hepatitis C | 0.01 | 0.06 |
| Fructose and mannose metabolism | 0.02 | 0.05 |
| Huntington's disease | 0.02 | 0.06 |
| Serotonergic synapse | 0.04 | 0.04 |
| Cholinergic synapse | 0 | 0.09 |
| NF-kappa B signaling pathway | 0.01 | 0.08 |
| Salmonella infection | 0.07 | 0.02 |
| Cell cycle | 0 | 0.1 |
| GABAergic synapse | 0.05 | 0.05 |
| Natural killer cell mediated cytotoxicity | 0 | 0.1 |
| Neuroactive ligand-receptor interaction | 0.08 | 0.02 |
| Nicotinate and nicotinamide metabolism | 0.02 | 0.09 |
| Oocyte meiosis | 0.05 | 0.06 |
| Antigen processing and presentation | 0 | 0.12 |
| Dilated cardiomyopathy | 0.01 | 0.11 |
| Glycerophospholipid metabolism | 0 | 0.12 |
| HIF-1 signaling pathway | 0 | 0.12 |
| Leukocyte transendothelial migration | 0 | 0.12 |
| Colorectal cancer | 0.01 | 0.12 |
| Influenza A | 0 | 0.14 |
| Retrograde endocannabinoid signaling | 0.03 | 0.11 |
| Leishmaniasis | 0 | 0.16 |
| Apoptosis | 0 | 0.17 |
| Herpes simplex infection | 0 | 0.18 |
| Gastric acid secretion | 0.01 | 0.18 |
| RIG-I-like receptor signaling pathway | 0 | 0.19 |
| African trypanosomiasis | 0.01 | 0.2 |
| Cocaine addiction | 0.04 | 0.17 |
| Adipocytokine signaling pathway | 0 | 0.22 |
| Long-term depression | 0.03 | 0.19 |
| Osteoclast differentiation | 0.01 | 0.21 |
| Tight junction | 0 | 0.22 |
| Amyotrophic lateral sclerosis (ALS) | 0.05 | 0.17 |
| Progesterone-mediated oocyte maturation | 0.02 | 0.21 |
| mTOR signaling pathway | 0 | 0.23 |
| Prion diseases | 0.01 | 0.22 |
| Bacterial invasion of epithelial cells | 0.01 | 0.23 |
| Carbohydrate digestion and absorption | 0 | 0.25 |
| Pentose phosphate pathway | 0.25 | 0.03 |
| Glioma | 0 | 0.29 |
| Melanoma | 0.02 | 0.27 |
| Small cell lung cancer | 0.02 | 0.27 |
| B cell receptor signaling pathway | 0.03 | 0.27 |
| Inositol phosphate metabolism | 0.04 | 0.27 |
| ECM-receptor interaction | 0 | 0.33 |
| Bladder cancer | 0.04 | 0.3 |
| Alzheimer's disease | 0 | 0.36 |
| Amino sugar and nucleotide sugar metabolism | 0 | 0.38 |
| Morphine addiction | 0.01 | 0.41 |
| Fc gamma R-mediated phagocytosis | 0 | 0.43 |
| Axon guidance | 0 | 0.46 |
| Insulin signaling pathway | 0.01 | 0.49 |
| Pertussis | 0 | 0.52 |
| Thyroid cancer | 0.02 | 0.52 |
| Wnt signaling pathway | 0 | 0.55 |
| Fc epsilon RI signaling pathway | 0.02 | 0.57 |
| Glycosphingolipid biosynthesis - lacto and neolacto series | 0.62 | 0 |
| Neurotrophin signaling pathway | 0 | 0.62 |
| TGF-beta signaling pathway | 0 | 0.63 |
| Toxoplasmosis | 0 | 0.66 |
| Endometrial cancer | 0 | 0.68 |
| NOD-like receptor signaling pathway | 0 | 0.71 |
| Endocrine and other factor-regulated calcium reabsorption | 0.03 | 0.71 |
| VEGF signaling pathway | 0.05 | 0.72 |
| ErbB signaling pathway | 0 | 0.92 |
